# PROTOCOL: Evaluating the application and effectiveness of precision teaching: A systematic review and meta‐analysis

**DOI:** 10.1002/cl2.1317

**Published:** 2023-03-19

**Authors:** Athanasios Vostanis, Paul A. Thompson, Ciara Padden, Konstantinos Rizos, Peter E. Langdon

**Affiliations:** ^1^ Tizard Centre University of Kent Canterbury UK; ^2^ Centre for Educational Development, Appraisal and Research University of Warwick Coventry UK; ^3^ Forest Bridge School Maidenhead UK; ^4^ Centre for Educational Development, Appraisal and Research, and Centre for Mental Health and Wellbeing Research University of Warwick Coventry UK

**Keywords:** behavioral agility, behavioral fluency, REAPS, RESA, standard celeration chart

## Abstract

Precision Teaching is a behavior measurement system that emphasizes the development of behavioral repertoires and utilizes Standard Celeration Charts as its primary tool. This system has been applied across various areas, including mainstream and special education, and has successfully improved academic, motor, communication, and other skills. While previous systematic reviews have highlighted important aspects of Precision Teaching, a more comprehensive evaluation is needed to consider all its different applications and recent developments in conceptualizing it. Therefore, this systematic review and meta‐analysis will assess the effectiveness of Precision Teaching in accelerating human behavior, identify all the areas of its application, and review the technical aspects of its implementation. The review aims to provide a comprehensive understanding of the system and its potential benefits for individuals in different settings.

## 1 BACKGROUND

### 1.1 The problem, condition, or issue

Precision Teaching has been recently evaluated in terms of its critical features (Evans et al., 2021). As a result, a synthesized definition was constructed along with a detailed concept and process analysis. As this conceptualization is recent, there has not been a thorough evaluation of the Precision Teaching literature against the modern criteria set by the field.

### 1.2 The intervention

Precision teaching is a system for precisely defining, measuring, recording, and analyzing behavior change across time that has emerged from the field of the experimental analysis of behavior (Kubina & Yurich, 2012). It has been applied to mainstream and special education (Beverley et al., 2016; Brady & Kubina, 2010; Chiesa & Robertson, 2000; Datchuk & Kubina, 2014; Greene et al., 2018; Johnson & Street, 2012; Lin & Kubina, 2015), and areas such as sports (Lokke et al., 2008; Pocock et al., 2010), traumatic brain injury (Chapman et al., 2005; Kubina et al., 2000), and private events, such as thoughts (Calkin, 2009; Patterson & McDowell, 2009).

### 1.3 How the intervention might work

Precision teaching has been described as a system focused on developing behavioral repertoires through precisely analyzing behavior change across time. Its precise approach helps practitioners provide the support that leads to accelerated outcomes and avoids prolonged periods of slow or no progression. Precision teaching uses a five‐step framework: pinpoint, practice, chart, decide, and try again (Evans et al., 2021). In the pinpoint phase, movement cycles and learning channels are used to pinpoint behavior. In other words, the skills or behaviors to be targeted are specified so improvements can be accurately measured. In the practice phase, instruction is arranged to promote the acceleration of the targeted skills. In the charting phase, dimensional behavior measures are used (e.g., frequency per minute) and a family of standardized graphical displays, known as the standard celeration charts (Calkin, 2005). In other words, a set of standardized displays is used to graph the data and monitor progress. In the decide phase, outcomes are evaluated, and practitioners engage in problem‐solving, if necessary. Finally, in the try‐again phase, remedial strategies are applied, and their effect is evaluated in a recursive manner (Evans et al., 2021).

### 1.4 Why it is important to do this review

Existing reviews have focused on different aspects of Precision Teaching but not in a manner that would capture the whole corpus of the Precision Teaching literature and make possible the evaluation of the field in terms of its various applications and effectiveness (Cihon, 2007; Doughty et al., 2004; Gist & Bulla, 2022; Heinicke et al., 2010; Martinho et al., 2022; Tiernan et al., 2022; Quigley et al., 2018; Ramey et al., 2016; Stocker et al., 2019). Specifically, Cihon (2007) focused on Precision Teaching's effectiveness in developing verbal behavior. Three reviews focused on the concept of behavioral fluency, which has emerged from the field of Precision Teaching (Doughty et al., 2004; Heinicke et al., 2010; Stocker et al., 2019). Quigley et al. (2018) focused on using a flashcards procedure that emerged from Precision teaching, known as SAFMEDS (Say All Fast a Minute Each Day Shuffled), to produce fluent responses. Ramey et al. (2016) focused on the application of Precision Teaching to supporting people with intellectual and developmental disabilities, while Martinho et al. (2022) focused on the effect of Precision Teaching and fluency training in supporting autistic individuals. Gist and Bulla (2022) focused on the combination of Precision Teaching with fluency‐building activities. Finally, another recent review focused on Precision Teaching's effectiveness when focused on academic skills (Tiernan et al., 2022). Although each review has produced important information about Precision Teaching and behavioral fluency, they have all used different definitions of Precision Teaching, which could have potentially led to inconsistent results. Also, none of them has thoroughly evaluated the Precision Teaching literature while considering the most recent standards set by the field (Evans et al., 2021).

This study links to current legislation and policy focusing on evidence‐informed approaches in mainstream and special education (Decuypere et al., 2011; Department of Education [DfE]; Lingard, 2013). Although it will also examine areas outside of education, it should provide meaningful information about how Precision Teaching has been applied and its effect across different skills and age groups. However, it is most likely to have an impact on policies focused on the use of teaching methods within schools and, broadly, the field of education and behavior analysis.

## 2 OBJECTIVES

This study will aim to answer a primary research question and a series of secondary questions. Specifically, the primary research question is whether Precision Teaching is an effective system for interventions aiming to increase human behavior.

Secondary research questions include:

1. In what areas and with what range of participants has Precision Teaching been applied?
2. What components of the Precision Teaching system are typically used and reported in applied studies?

## 3 METHODS

### 3.1 Criteria for considering studies for this review

#### 3.1.1 Types of studies

We will include between‐group studies, randomized controlled trials, or quasi‐experimental studies involving a nonexposed control group or an attention‐control group. We will also include studies using time series and case studies, including quasi‐experimental baseline‐intervention (A‐B) designs. Studies will be excluded if they do not report baseline or pre‐assessment data unless an alternating treatments design is used.

#### 3.1.2 Types of participants

We will include studies that involve children, adolescents, or adults with no age restrictions. Participants with any diagnosis and from any background will be included in the studies that satisfy our eligibility criteria.

#### 3.1.3 Types of interventions

To be included in the review, studies will have to pass two rounds of screening against a series of general and specific eligibility criteria without placing a limit to their year of publication. Specifically, in the first round of review, studies will have to:

1. Be peer‐reviewed
2. Be written in English
3. Include a PT intervention or at least one of its critical components, as defined by Evans et al. (2021).
4. Included measures of behavior change
5. Include a baseline or pre‐test measure.

In the second round of review, studies will be evaluated against a set of more specific criteria. Evans et al. (2021) specified six critical features of Precision Teaching. Those critical features will allow us to evaluate whether studies meet the criteria to be considered representative of Precision Teaching. Such a step is necessary as the term has been used inconsistently in the literature. In this study, we will use five of the six criteria. Specifically, to be included, studies will need to:

1. Focus on accelerating behavioral repertoires.
2. Use definitions that allow us to identify the exact behaviors targeted.
3. Use continuous observation.
4. Use dimensional measurements.
5. Use (or explicitly mention using) the family of standard celeration charts.

Studies using other visual displays or standard celeration chart “look‐a‐likes” will be excluded as the chart's standardization is considered fundamental in precision teaching (Evans et al., 2021). The studies will be included if authors report using different displays with their participants but the standard celeration charts for their analysis. The final feature Evans et al. (2021) describes is making timely and effective decisions. We decided that this component was not entirely relevant to academic studies where the conditions might have been pre‐determined to protect internal validity. Therefore, we will not evaluate studies against this criterion.

#### 3.1.4 Types of outcome measures

Outcomes will not affect eligibility but will include the amount of behavior change across time measured via direct observations, standardized assessments, or curriculum assessments. Depending on how behavior change was assessed, outcomes might include data on frequency, duration, latency, change on average performance, change in standard scores, and similar measures.

##### Primary outcomes

Amount of behavior change across time.

##### Secondary outcomes

Evaluation of the by‐products of fluent performance defined as maintenance/retention, endurance, stability, application/generalization, and generativity.

#### 3.1.5 Duration of follow‐up

We will look to summarize changes at follow‐up across studies, including the duration of follow‐up used by authors within the included studies. Where multiple follow‐up points are used, we will extract the data in their entirety. We anticipate this will vary across studies but is assumed to be at least one week.

#### 3.1.6 Types of settings

We will not be placing any restrictions on the settings.

### 3.2 Search methods for identification of studies

We will conduct systematic searches by entering keyword combinations into a series of databases and platforms. Regarding databases, we will use EBSCO information services to access APA PsycINFO, ERIC, and British Education Index, while we will also access Scopus through Elsevier and we will also access Pubmed. Regarding platforms, we will use Web of Science's core collection. We will not set criteria based on the population, comparison, or outcomes to include as many relevant studies as possible.

We will also hand‐search journals that have historically published Precision Teaching literature. Specifically, the Journal of Precision Teaching and Celeration, the European Journal of Behavior Analysis, the Journal of Behavioral Education, Behavioral Interventions, Educational Psychology in Practice, Irish Educational Studies, and TEACHING Exceptional Children. We also check for potentially relevant articles at www.fluency.org, a website with a list of Precision Teaching articles and other resources. Finally, we will engage in forward and backward citation searching.

#### 3.2.1 Electronic searches

The keyword combinations will apply, where possible, to the title, abstract, full text, descriptors, exact descriptors, and identifiers and will be: “Precision Teach*” OR Frequency‐Build* OR Fluency‐Build* OR “Frequency Building to a Performance Criterion” OR FBPC OR SAFMEDS OR “Say All Fast a Minute Every Day Shuffl*” OR Component Composite OR Element Compound OR “Learning Channel*” OR Standard Celeration Chart* OR Big 6.

#### 3.2.2 Searching other resources

We are expecting more than 150 studies to be included in this review. Therefore, due to the volume of articles that will need to be processed, we decided that it would not be possible to include gray literature and focus only on studies published within journals that operate a peer‐review process.

### 3.3 Data collection and analysis

#### 3.3.1 Description of methods used in primary research

Studies will, in most cases, use time‐series designs such as Baseline‐Intervention (A‐B) designs, multiple baseline across participants designs, reversal designs, or alternating treatments designs. Studies that meet the eligibility criteria will include, at the very minimum, a baseline and intervention phase, unless an alternating treatments design is used, which can be implemented without a baseline condition, although it is not optimal. Some studies will use between‐group designs, either quasi‐experimental or randomized controlled trials. Quasi‐experimental designs will involve one group and pre‐post‐assessments of intervention outcomes or two non‐randomized groups. All studies will use a Precision Teaching framework as an absolute minimum along with additional educational or behavioral strategies such as frequency building to a performance criterion (Datchuk et al., 2015), Say All Fast a Minute Each Day Shuffled (Quigley et al., 2018), Talk‐Aloud Problem Solving (Dembek & Kubina, 2018), and self‐management techniques such as measuring positive and negative “inner” behavior (Patterson & McDowell, 2009).

#### 3.3.2 Selection of studies

First, one of the authors will screen studies against the eligibility criteria by reading the title and abstract. Once irrelevant studies are excluded, they will engage in two rounds of full‐text screening against each set of eligibility criteria respectively, which will lead to the final pool of relevant papers. A second author will allow us to calculate interrater agreement by independently repeating this process with a randomly selected sample that will consist of at least 20% of the studies for title‐abstract and two rounds of full‐text screening. A minimum criterion of 90% agreement will be set. If that criterion is not met, then an additional sample of 20% will be provided for evaluation. Also, disagreements will be discussed between the two authors, and if a verdict is not reached, one of the remaining authors will be involved to decide whether a study will be included or excluded.

#### 3.3.3 Data extraction and management

One of the authors will extract the data from the studies and add them to the relevant data extraction table using Microsoft Excel™. A second author will independently repeat the process with a randomly selected sample that will consist of 40% of the studies, and an interrater agreement will be calculated. A minimum criterion of 90% agreement will be set. If that criterion is not met, then an additional sample of 40% will be provided for evaluation. Disagreements will be discussed between the two authors, and if no verdict is reached, one of the remaining authors will decide the course of action.

A minimum of the following data, divided into primary and secondary, will be extracted from each included study.

Primary data will include the following:

- Country where studies took place
- Studies' aims
- Targeted areas
- Participant information including age, sex, ethnicity, diagnoses, and levels of attrition
- Setting where studies were conducted
- Experimental design
- Targeted behaviors
- Measures of social validity
- Intervention information
- Intensity of intervention
- Reported outcomes

Secondary data will include the following:

- Learning channels used
- Visual displays presented in the articles
- Frequency aims specified
- Duration of counting times (timings)
- Precision Teaching metrics used, such as celeration or bounce
- Mastery assessments on retention, endurance, stability, application, and generalization (RESAG).

#### 3.3.4 Assessment of risk of bias in included studies

We will use a series of tools to evaluate the quality of the studies included in this review. For studies using time‐series designs, we will use the evaluative method described by Reichow et al. (2008). The evaluative method is based on a series of primary and secondary indicators that allow the evaluator to produce an overall quality rating for each study. The tool offers detailed guidance about how each indicator should be scored, but it has also been adapted to better fit the purpose of other reviews (Brady et al., 2019; Tomlinson et al., 2018). To make sure that the tool is tailored to the purpose of the study, we will make four adaptations to the original tool. First, since some studies include typically developing individuals, we will code those without expecting them to explicitly state this information in line with other systematic reviews (Brady et al., 2019). What is more, due to the different types of studies that will be included in this review, we will score the first quality indicator (i.e., Participants) as high quality if it meets 3/3 or 4/4 criteria. That way, studies that did not use standardized assessments will still be eligible for a high‐quality rating. In line with this decision, we will score this indicator as Acceptable if 2/3 or 3/4 criteria are present. Second, we will use a five‐scale rating of overall quality instead of the original three‐scaled one to improve the sensitivity of our assessment. The five‐scale overall quality rating has been successfully used in other systematic reviews (Brady et al., 2019; Tomlinson et al., 2018). Third, we will adapt the baseline quality indicator. Instead of relying solely on visual analysis, we will also use an effect size that evaluates baseline stability, such as the TAU‐U. Specifically, baseline data will be considered unstable if they produce a TAU‐U score of more than 0.40 (Parker et al., 2011). Fourth, we will adapt the criteria to account for alternating treatment designs, as the original scale provides no suggestion on how to rate studies using this experimental design. Specifically, if a study uses an alternating treatments design without a baseline, that indicator will be scored as weak. Although these designs have historically been used without a baseline, we decided it was essential to have a baseline to evaluate the effectiveness of outcomes. Also, to score a high‐quality rating with these designs, 100% of graphs will need to have (a) stable data, (b) no more than 25% of data points overlapping between the data paths, and (c) either a data path showing a treatment effect that is visibly distant from the rest of the data paths or an intervention data path that is visibly distant from a control condition data path. To score a high‐quality rating, 100% of graphs will need to meet all three criteria. For an acceptable quality rating, 66% of the graphs will need to meet at least two of the three criteria mentioned above. For an unacceptable rating, less than 66% of the graphs will meet at least two of the three criteria.

For studies using other non‐randomized between‐group designs, or experiments such as randomized controlled trials, we will use the evaluative method's rubric for group studies accompanied by the checklists developed by the JBI Institute (formerly known as Joanna Briggs Institute; Tufanaru et al., 2020). We made this decision because the JBI checklists offer additional guidance on evaluating studies using between‐groups quasi‐experimental designs or randomized controlled trials.

One of the authors will evaluate the studies against the quality assessment tools. A second author will independently evaluate the quality of a randomly selected sample that will consist of 40% of the studies, and an interrater agreement will be calculated. A minimum criterion of 90% agreement will be set. If that criterion is not met, then an additional sample of 40% will be provided for evaluation. Disagreements will be discussed between the two authors, and if no verdict is reached, one of the remaining authors will decide the course of action.

#### 3.3.5 Measures of treatment effect

We will engage in a narrative synthesis using all studies, regardless of quality rating, that will consider behavior change measured via direct observations, standardized assessments, or curriculum assessments. Depending on how behavior change was assessed, outcomes might include data on frequency, duration, latency, change on average performance, change in standard scores, and similar measures. These data are measures of treatment effect.

Meta‐regression will be conducted with random‐effects models with robust variance estimation, including both hierarchical and correlated effects structures to allow for the inclusion of dependent effect sizes to evaluate the effectiveness of Precision Teaching across various skill areas and domains. Extracted data will be continuous, and where possible, we will calculate the Hedge's *g* and use the standardized mean difference (SMD) with a 95% confidence interval. A substantial proportion of extracted data is likely to come from time series designs. In these cases, we will use the Between‐Case Standardized Mean Difference with a 95% confidence interval (Shadish et al., 2014). This effect size produces similar parameters to Hedges' *g*. In addition, we will calculate a non‐parametric effect size, such as the TAU‐U or Baseline‐Corrected TAU, that focuses on the overlap between phases while evaluating the baseline trend (Parker et al., 2011; Tarlow, 2017).

We will include all studies in the meta‐analysis, irrespective of their quality. To that end, we will conduct a meta‐regression analysis, including study quality as a metric.

#### 3.3.6 Unit of analysis issues

We will analyze data at the participant level and avoid double‐counting participants. We do not anticipate the inclusion of any cluster‐randomized or cross‐over controlled trials. Still, should they be included, we will take clustering into account within our analysis to avoid incorrectly estimating the treatment effect. Studies with more than two groups will be included, but we will avoid double‐counting by combining intervention groups if possible or excluding intervention groups that are not of interest. We will conduct a separate analysis of outcome data following the completion of treatment (short‐term outcomes) and at follow‐up (medium‐term outcomes) where possible.

#### 3.3.7 Criteria for determination of independent findings

We will extract data on behavior frequency, duration or latency, change on average performance, change in standard scores, and similar measures. We will likely encounter multiple reports from the same study where behavior frequency, duration, or latency is reported for the same participants over time, and all data will be extracted. To deal with the dependency introduced, we will use meta‐regression with robust variance estimation, including both hierarchical and correlated effects structures to allow for the inclusion of dependent data.

#### 3.3.8 Dealing with missing data

We will analyze the available data. Where we encounter missing data, we will attempt to contact authors and gain access to data if possible. We will consider the impact of missing data on our analysis in the discussion of our findings and make a judgment about the risk of bias arising from missing data.

#### 3.3.9 Assessment of heterogeneity

We will examine heterogeneity using *I* ^2^ and report the prediction interval. This has been chosen rather than Cochrane's *Q* as it allows for the quantification of the effect of heterogeneity and allows for an estimate of the degree of inconsistency in the results, and is not dependent upon the number of included studies. We will consider heterogeneity without our narrative synthesis with reference to our findings arising from the quality appraisal.

#### 3.3.10 Assessment of reporting biases

We will complete a quality assessment of the methodology of included studies and report our findings with a focus on describing biases. Further, if possible, we will remove outliers from any meta‐analysis and recalculate weighted mean effect sizes. Publication bias will be assessed graphically using funnel plots, plotting summary effect sizes against standard error. Where possible, Fail‐Safe N will be used to assess the impact of bias. A figure exceeding 5*n* + 10 will be considered indicative of results robust to publication bias.

#### 3.3.11 Data synthesis

Analyses will primarily be conducted using SPSS and R using the metafor, clubSandwich, and/or robumeta packages. Random‐effects models with robust variance estimation, including both hierarchical and correlated effects structures, will be used, allowing for the inclusion of dependent effect sizes within meta‐regression (Pustejovsky & Tipton, 2022). Data will be extracted from randomized controlled trials and studies involving at least two groups of participants.

For studies employing time series designs, we will calculate the between‐case standardized mean difference set at a 95% confidence interval (Shadish et al., 2014). This effect size provides common parameters to that of Hedges' *g* (Shadish et al., 2014). Therefore, due to the inclusion of studies using time series‐ and between‐group designs in this review, it was considered appropriate to use this effect size to improve consistency in interpreting results. In addition, we will calculate a non‐parametric effect size, such as the TAU‐U or Baseline‐Corrected TAU, that focuses on the overlap between phases while evaluating the baseline trend (Parker et al., 2011; Tarlow, 2017). The data will be extracted from the graphs using appropriate digitizing software.

#### 3.3.12 Subgroup analysis and investigation of heterogeneity

A sub‐group analysis will be conducted to evaluate any observed heterogeneity further. A Galbraith plot will also be used to identify potential outliers, and a leave‐one‐out analysis will be conducted to calculate an aggregate effect size without outliers. Also, Prediction Intervals will be calculated to provide information about the dispersion of true effects around the weighted mean (IntHout et al., 2016). Finally, a one‐study‐removed forest plot will be created to evaluate further each study's impact on the weighted mean and heterogeneity.

#### 3.3.13 Sensitivity analysis

Due to the expected heterogeneity, a meta‐regression will be conducted, including metrics such as the study quality, number of participants, participants' sex, and duration of practice, amongst others.

#### 3.3.14 Treatment of qualitative research

We do not plan to include qualitative research.

#### 3.3.15 Summary of findings and assessment of the certainty of the evidence

We will include an overall summary of our findings with reference to our quality appraisal, bias, and associated heterogeneity. We will focus on the key outcome, which will be the amount of behavior change across time. Our assessment of the body of evidence will include a quality appraisal tool designed specifically for *n* = 1 designs and another tool for quasi‐experimental designs and randomized control trials.

## CONTRIBUTIONS OF AUTHORS

- Content: Athanasios Vostanis and Konstantinos Rizos.
- Systematic review methods: Peter E. Langdon, Athanasios Vostanis, Ciara Padden
- Statistical analysis: Paul A. Thompson, Peter E. Langdon, and Athanasios Vostanis.
- Information retrieval: Athanasios Vostanis and Konstantinos Rizos.

## DECLARATIONS OF INTEREST

All the authors, apart from Dr. Thompson, have published research on Precision Teaching. There is no other conflict of interest to report.

## PRELIMINARY TIMEFRAME

The approximate date for submission of the systematic review: February 2024

## PLANS FOR UPDATING THIS REVIEW

Athanasios Vostanis will be responsible for updating this review every 5–7 years.

## SOURCES OF SUPPORT

**Internal sources**

- Internal Funding, UK

No financial support to declare

**External sources**

- External Funding, UK

No financial support to declare.
